# Response to ‘OPN gene polymorphisms influence the risk of knee OA and OPN levels in synovial fluid in a Chinese population’

**DOI:** 10.1186/s13075-014-0433-0

**Published:** 2014-09-26

**Authors:** Chao Zeng, Shu-guang Gao, Guang-hua Lei

**Affiliations:** Department of Orthopaedics, Xiangya Hospital, Central South University, #87 Xiangya Road, Changsha, Hunan Province 410008 China

We read with great interest the article by Jiang and colleagues in a recent issue of *Arthritis Research & Therapy* related to osteopontin (OPN) gene polymorphisms influencing the risk of knee osteoarthritis (OA) and the OPN levels in synovial fluid in a Chinese population [1]. The authors showed that the thrombin-cleaved osteopontin (OPN-N) levels in synovial fluid were positively correlated to the radiographic severity of OA, which was consistent with our result [2]. In addition, the group indicated that OPN-N levels (but not the full-length OPN levels) in synovial fluid were significantly lower in certain OPN gene polymorphisms (–443TT and –66GG). We believe this result was very intriguing to lots of readers. However, the discussion of this specific part was unclear and deserves more attention.

For the –443 locus, the authors suggested that –443 T had a lower promoter activity that could regulate the OPN gene transcript activity, thus influencing OPN expression. Similarly, for the –66 locus, overexpression of specificity protein 1 induced higher promoter activation on a plasmid carrying the T allele compared with the equivalent plasmid carrying the G allele that was used to explain their findings. In other words, alleles at polymorphic sites in the OPN gene influence the OPN level by influencing the level of promoter activity and characterizing their ability to bind transcription factors that had been proposed by the authors. Perhaps most puzzling of all, these explanations actually conflicted with their findings – certain OPN gene polymorphisms of the two loci (–66, –443) were not associated with full-length OPN, but with OPN-N.

Almost at the end of the discussion, the authors postulate that the influence of OPN polymorphisms on risk and severity of OA is very probably due to its effect on the synthesis of the active OPN protein. Combined with the result of the study, we speculate that the authors mean that certain OPN polymorphisms could lead to active OPN protein that can easily be cut by thrombin. However, the loci (–66, –443) are all in the human OPN promoter, but not the coding sequence. Theoretically, a promoter is a region of DNA that initiates transcription of a particular gene that could influence the expression of this gene but may not have an effect on the properties of the corresponding protein.

So what are the possible reasons that could explain the high OPN-N level in OA patients? Liu and coworkers suggested that significant higher expression of thrombin was observed in OA synovial fibroblasts than in normal synovial fibroblasts [3]. Ohba and colleagues found high levels of thrombin activity in rheumatoid arthritis [4]. Myles and Leung indicated that OPN cleavage by thrombin was dependent on both anion-binding exosites in thrombin that determined the rates and specificity of thrombin proteolysis [5]. Alongside these potential reasons (higher expression or activity of thrombin), Jones and colleagues found that antithrombin activity is selectively depressed in rheumatoid arthritis synovial fibroblasts [6].

Above all, we respect the great contribution of the authors and have no doubt that the results of the data are accurate. We are just curious about the association between certain OPN gene polymorphisms and OPN-N (not full-length OPN). We would be very grateful if the authors could discuss this association more clearly. The association between certain OPN gene polymorphisms and OPN-N also deserves exploration in further studies.

## Abbreviations

OA: Osteoarthritis
OPN: Osteopontin
OPN-N: Thrombin-cleaved osteopontin
